# Electrospun Amorphous Solid Dispersions with Lopinavir and Ritonavir for Improved Solubility and Dissolution Rate

**DOI:** 10.3390/nano14191569

**Published:** 2024-09-28

**Authors:** Ewelina Łyszczarz, Oskar Sosna, Justyna Srebro, Aleksandra Rezka, Dorota Majda, Aleksander Mendyk

**Affiliations:** 1 Department of Pharmaceutical Technology and Biopharmaceutics, Faculty of Pharmacy, Jagiellonian University Medical College, Medyczna 9, 30-688 Cracow, Poland; 2 Doctoral School of Medicinal and Health Sciences, Jagiellonian University Medical College, Św. Łazarza 16, 31-530 Cracow, Poland; 3 Department of Chemical Technology, Faculty of Chemistry, Jagiellonian University, Gronostajowa 2, 30-387 Cracow, Poland

**Keywords:** lopinavir, ritonavir, poorly soluble drug, electrospinning, amorphous solid dispersion, dissolution enhancement

## Abstract

Lopinavir (LPV) and ritonavir (RTV) are two of the essential antiretroviral active pharmaceutical ingredients (APIs) characterized by poor solubility. Hence, attempts have been made to improve both their solubility and dissolution rate. One of the most effective approaches used for this purpose is to prepare amorphous solid dispersions (ASDs). To our best knowledge, this is the first attempt aimed at developing ASDs via the electrospinning technique in the form of fibers containing LPV and RTV. In particular, the impact of the various polymeric carriers, i.e., Kollidon K30 (PVP), Kollidon VA64 (KVA), and Eudragit^®^ E100 (E100), as well as the drug content as a result of the LPV and RTV amorphization were investigated. The characterization of the electrospun fibers included microscopic, DSC, and XRD analyses, the assessment of their wettability, and equilibrium solubility and dissolution studies. The application of the electrospinning process led to the full amorphization of both the APIs, regardless of the drug content and the type of polymer matrix used. The utilization of E100 as a polymeric carrier for LPV and KVA for RTV, despite the beads-on-string morphology, had a favorable impact on the equilibrium solubility and dissolution rate. The results showed that the electrospinning method can be successfully used to manufacture ASDs with poorly soluble APIs.

## 1. Introduction

The poor bioavailability due to the poor solubility and low dissolution rate of APIs remains a significant challenge in developing oral dosage drug forms. This is especially critical for active substances belonging to class II and IV of the Biopharmaceutics Classification System (BCS), thus characterized by low solubility [1]. Examples include certain antiviral drugs such as ritonavir (RTV) and lopinavir (LPV), which are first- and second-generation human immunodeficiency virus 1 (HIV-1) protease inhibitors. To improve the solubility and dissolution rate of LPV and RTV, several methods have been proposed, such as liquisolid technology [2], the formation of solid lipid nanoparticles [3], co-crystals [4], nanocrystals [5], hybridized nanoamorphous micellar dispersion [6], as well as the preparation of solid dispersions via solvent evaporation [7,8], hot-melt extrusion [7,9,10,11], and freeze drying [12].

Solid dispersion (SD) is a commonly employed technique, resulting in the enhancement of drug dissolution and bioavailability, primarily through the reduction in particle size, the decrease in the crystallinity degree, the reduction in tendency towards agglomeration, and the enhancement of wettability [13,14]. So far, five generations of solid dispersions have been described in the literature [15]. Currently, the most common approach is to disperse hydrophobic APIs in a hydrophilic carrier [13,16]. The choice of the appropriate polymer has an impact on the enhancement of dissolution, bioavailability, and stability of the drug. Polymers used for the preparation of SD include polyvinylpyrrolidone (PVP), polyvinylpyrrolidone-co-vinyl acetate (PVP-VA), polyvinyl alcohol (PVA), polymethacrylates, hydroxypropylmethyl cellulose (HPMC), hydroxypropylcellulose (HPC), and hydroxypropylmethyl cellulose acetate succinate (HPMC-AS). Solid dispersions can be prepared using various methods, such as melting or hot-melt extrusion [9,17], ball milling [18,19,20], supercritical fluid technology [21], or solvent evaporation [20,22], especially useful in preliminary screening tests. Recently, among the solvent evaporation methods, the electrospinning technique has been gaining popularity [15,23,24,25].

Electrospinning involves the production of polymer fibers in an electrostatic field. The application of high voltage leads to stretching and elongating of the polymeric solution. The electrified fluid jet is ejected from the apex of the Taylor cone and accelerated by the electric field. The solvent evaporates quickly, and the fibers are formed when the jet reaches the collector. This process leads to the swift formation of fibrous mats, which reduces the mobility of drug molecules within the polymer fibers and prevents the formation of a crystalline lattice. It leads to a random distribution of drug molecules within the formed drug–polymer fibers [26,27]. The formation of electrospun drug–polymer matrices leads to an increase in the surface-to-volume ratio, which affects the dissolution performance. Moreover, the high degree of mixing between the API and the polymer, which is difficult to obtain via powder blending, combined with a high evaporation rate, facilitates the formation of physically stable amorphous solid dispersions. Due to that, this technique is perfect for obtaining the amorphous solid dispersion of poorly water-soluble active substances [15].

This research aimed to obtain an amorphous solid dispersion (ASD) containing LPV or RTV as the model drugs via the electrospinning method. The impact of the various polymeric carriers, i.e., polyvinylpyrrolidone, polyvinylpyrrolidone-co-vinyl acetate, and Eudragit^®^ E100, as well as the drug content on the fibers’ morphology, wettability, equilibrium solubility, and dissolution rate of the APIs was assessed. To confirm the amorphous structure of the electrospun fibers, X-ray diffraction (XRD) and differential scanning calorimetry (DSC) were used.

## 2. Materials and Methods

### 2.1. Materials

Lopinavir (LPV, (2*S*)-*N*-[(2*S*,4*S*,5*S*)-5-[[2-(2,6-dimethylphenoxy)acetyl]amino]-4-hydroxy-1,6-diphenylhexan-2-yl]-3-methyl-2-(2-oxo-1,3-diazinan-1-yl)butanamide) and ritonavir (RTV, 1,3-thiazol-5-ylmethyl *N*-[(2*S*,3*S*,5*S*)-3-hydroxy-5-[[(2*S*)-3-methyl-2-[[methyl-[(2-propan-2-yl-1,3-thiazol-4-yl)methyl]carbamoyl]amino]butanoyl]amino]-1,6-diphenylhexan-2-yl]carbamate) were purchased from Wuhan ChemNorm Biotech Co., Ltd., Wuhan, China. A cationic copolymer based on dimethylaminoethyl methacrylate, butyl methacrylate, and methyl methacrylate with a ratio of 2:1:1 (E100, Eudragit^®^ E100), kindly donated by Evonik Industries AG, Essen, Germany, poly(vinylpyrrolidone) (PVP, Kollidon K30), and poly(vinylpyrrolidone-vinyl acetate) (KVA, Kollidon VA64), both purchased from BASF, Ludwigshafen am Rhein, Germany, were used as polymeric matrices for ASDs. Polyoxyethylene 10 lauryl ether (Brij-35, HyperChem, Hangzhou, China) was used to prepare medium for the solubility and dissolution study. The water used in all tests was produced by an Elix 15UV Essential reverse osmosis system (Merck KGaA, Darmstadt, Germany). All other reagents were of analytical grade.

### 2.2. Preparation of Solutions for Electrospinning

Based on the preliminary optimization of a polymer concentration in placebo electrospun solutions, 30% *w/w* PVP, 35% *w/w* KVA, and 25% *w/w* E100 were selected for the preparation of ASDs with LPV and RTV by the electrospinning method. The solutions were prepared by dissolving the appropriate amount of polymer in 96% (*v/v*) ethanol with continuous stirring at 250 rpm using a Heidolph MR HeiTec magnetic stirrer (Schwabach, Germany). Raw LPV or RTV was added to the polymeric solution in different concentration ratios (Table 1) and mixed with a laboratory glass rod until the APIs were completely dissolved.

### 2.3. Electrospinning Process

A 20 mL syringe was filled with the electrospinning solution and mounted in a syringe pump (Ascor AP14, Ascor Med Sp. z o.o., Warsaw, Poland). An injection needle with a diameter of 0.6 mm was attached to the syringe through a silicone tube. Due to clogging of the needle during the process, it was necessary to increase its diameter to 0.7 mm for PVP30LPV10 and KVA35RTV5, 0.8 mm for PVP30LPV20 and E35RTV2.5, and 0.9 mm for E25RTV5. The needle was connected to the positive electrode of a high-voltage power supply (E-Fiber EF020 SKE Research Equipment^®^, Bollate, Italy). The grounding electrode was connected to a stationary collector (20 × 20 cm) wrapped in aluminum foil. The electrospinning process was carried out at room temperature and approx. 40% relative humidity (RH). The needle was charged with 30 kV, and the distance between the needle and the collector was 25 cm. The feeding rate of the spinning solution was set at 4 mL/h, and the process was conducted for 3 h.

### 2.4. Characterization of the ASDs

#### 2.4.1. Morphological Assessment

The morphology of the electrospun fibers containing LPV or RTV was examined using a Hitachi S-4700 (Tokyo, Japan) scanning electron microscope, due to its higher resolution. The accelerating voltage of the beam was equal to 20 kV. Small pieces of the fiber mats (approx. 0.5 × 0.5 cm) were placed on the SEM conductive adhesive tape previously glued to a specimen mount. The electrospun fibers were sputtered with gold. Microphotographs were taken at magnifications of 500×, 1000×, 2000×, 5000×, and 10,000×.

#### 2.4.2. Differential Scanning Calorimetry (DSC)

The thermodynamic properties of the raw LPV, raw RTV, polymers, and prepared electrospun fibers were examined using a Mettler-Toledo DSC 3+ System (Greifensee, Switzerland). A known weight of the tested sample was heated in an argon atmosphere (50 cm^3^/min) within the range of 293 K to 473 K at a heating rate of 10 K/min. Next, the samples were cooled down at a 10 K/min rate to room temperature and re-heated to 293 K in a second run. Measurements were performed in an aluminum pan with a pierced lid. Melting points were determined as the onset of the peak in the first run, while the temperature of the glass transition was determined in the second run.

#### 2.4.3. X-ray Diffraction (XRD)

The crystalline structure of the samples, i.e., raw LPV, raw RTV, polymers, and prepared electrospun fibers, was analyzed at an ambient temperature using a Philips PW1830 X-ray diffractometer (Amsterdam, The Netherlands) equipped with X’Pert Data Colletion version2.0e produced by PANalytical B.V. Diffraction patterns were collected over a 2θ range between 3° and 35° with a 5°/min step. Samples were tested as received.

#### 2.4.4. Wettability Study

The sessile drop technique was applied to determine the wettability of raw APIs and electrospun fibers with a DSA255 drop shape analyzer (Krüss, Hamburg, Germany). The droplet of the distilled water of volume equal to 2 µL was deposited on the surface of electrospun fibers as well as on the raw LPV and RTV. The fibers and APIs were compressed using an AtlasTM manual 15Ton hydraulic press (Specac, Kent, UK) with a load pressure of 2 tons applied for 30 s for each sample. All the measurements were carried out in triplicate.

#### 2.4.5. High-Performance Liquid Chromatography (HPLC)

LPV and RTV quantification was performed using the HPLC method. A Jasco LC-NetII/ADC (JASCO Corporation, Tokyo, Japan), equipped with a diode array detector, an integrated autosampler, and an InfinityLab Poroshell 120 EC-C18 column (100 × 4.6 mm, 4 µm particle size), Agilent, were utilized for analysis. The mobile phase was composed of water and acetonitrile (40:60 *v/v*) and the flow rate was 1.5 mL/min. The column was maintained at ambient temperature (approx. 295 K). The injection volume was set at 10 µL and the total run time was 3 min. The retention time of LPV and RTV was 1.7 and 2.0, respectively. The UV detection was carried out at λ = 210 nm for LPV and at λ = 240 nm for RTV. Linearity for both APIs was confirmed within the concentration range of 2.0–140.0 μg/mL (R^2^ = 0.9999).

#### 2.4.6. Drug Loading and Encapsulation Efficiency

Accurately weighed samples of prepared electrospun fibers were transferred into 25 mL flasks and dissolved in methanol. Afterward, the samples were filtered through a nylon 0.22 μm VWR^®^ syringe filter (Avantor, Radnor, PA, USA), and the amount of LPV and RTV was determined by HPLC.

The encapsulation efficiency (*EE*, %) of the electrospun fibers was determined based on the following equation:

(1) $$EE\%=\frac{Drug\;Loading\;}{Theoretical\;Drug\;Loading}$$

#### 2.4.7. Equilibrium Solubility

An excess of crystalline LPV and RTV and electrospun fibers with LPV or RTV was dispersed in 1 mL of 0.1 M hydrochloric acid (pH = 1.2), phosphate buffer (pH = 6.8), and 0.06 M polyoxyethylene 10 lauryl ether (Brij-35) (pH = 4.1). The suspensions were shaken at 500 rpm at ambient temperature (approx. 298 K) for 48 h using the IKA^®^ KS-130 shaker (Königswinter, Germany) until equilibrium was reached. The samples were filtered through a nylon 0.22 μm VWR^®^ syringe filter (Avantor). The samples were analyzed in triplicate using the HPLC method. The reported data represent the averages of three series of measurements with standard deviations (SDs).

#### 2.4.8. Dissolution Studies

The dissolution studies were performed in accordance with the USP 43-NF38 guidance for LPV/RTV tablets [28] using a pharmacopoeial apparatus type II (Hanson Vision G2 Elite 8, Chatsworth, CA, USA) equipped with a VisionG2 AutoPlus autosampler. The electrospun fibers were inserted into stainless-steel sinkers and transferred into 900 mL of 0.06 M polyoxyethylene 10 lauryl ether solution of pH 4.1 at 37 °C. The paddle rotation speed was 75 rpm. Filtered samples were withdrawn at 1, 3, 5, 10, 15, 30, 45, 60, and 90 min. The samples were filtered through a nylon 0.22 μm VWR^®^ syringe filter (Avantor) and analyzed using the HPLC method. The tests were carried out in triplicate, and the results represent the averages with their standard deviations (SDs).

## 3. Results and Discussion

The selection of a suitable polymer and solvent has an impact on electrospun fiber morphology [27]. In the electrospinning process, a drug substance can be dissolved or suspended in a polymeric solution [29,30]. In this study, it was assumed that solutions of LPV and RTV would be used, due to the possibility of preparing second-generation ASDs [15]. Hence, considering the physicochemical characteristics of LPV and RTV (Table 2), particularly the solubility data, absolute ethanol was selected as a solvent. Based on a preliminary study and literature data, polymers commonly used in the electrospinning process, i.e., PVP, KVA, and E100, were selected [15,24,25,31,32,33,34,35]. Their concentration in the ethanol solution was 30%, 35%, and 25%, respectively, despite the presence of fibers with bead-on-string morphology. The chosen polymer concentrations allowed the highest amount of the model drug substances to be incorporated.

### 3.1. Morphology of the Electrospun Fibers

**Figure 1:**
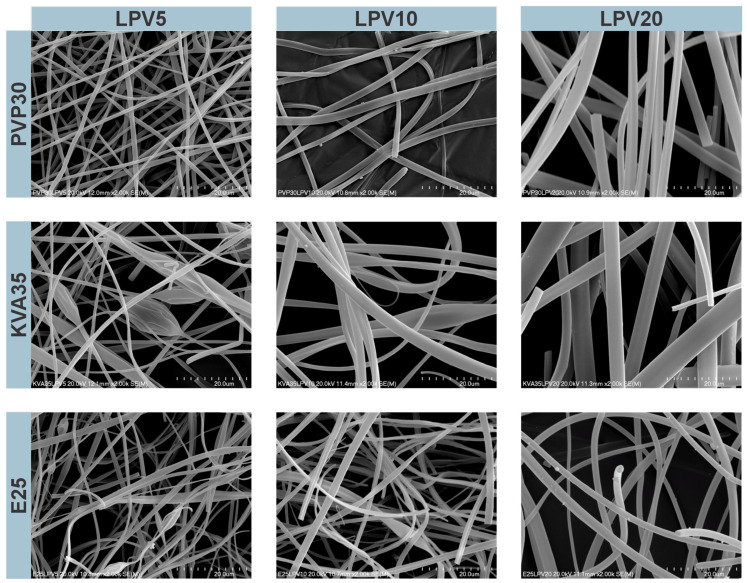
Scanning electron microscopic images of electrospun fibers with lopinavir (LPV), magnification 2000×.

**Figure 2:**
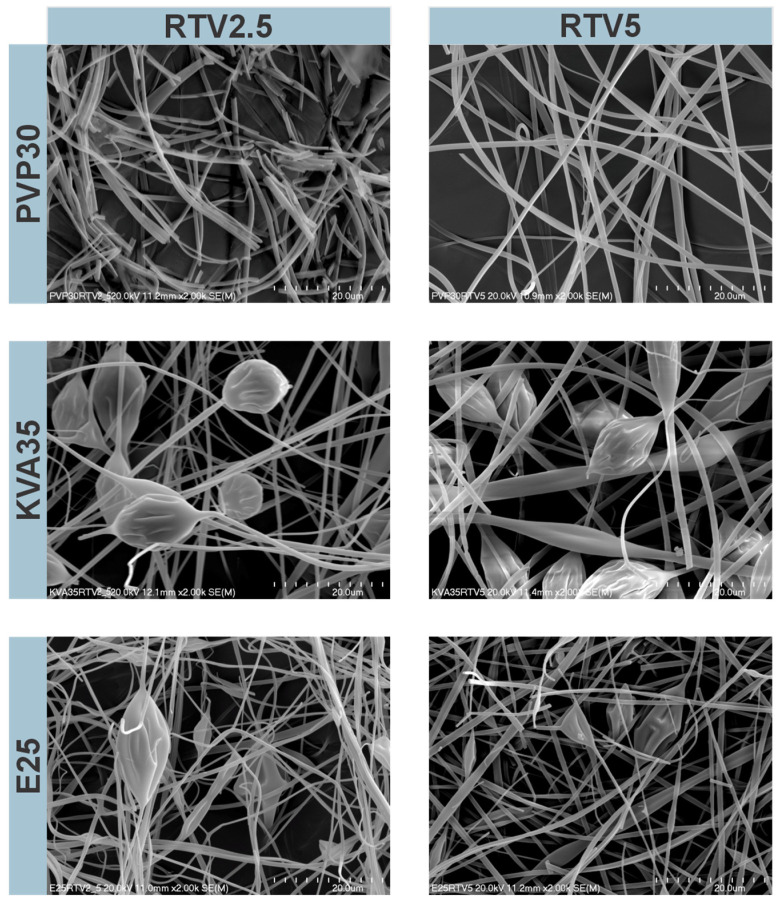
Scanning electron microscopic images of electrospun fibers with ritonavir (RTV), magnification 2000×.

Morphology and fiber thickness were analyzed based on the visual assessment and SEM images (Figure 1 and Figure 2). Differences in fiber morphology were observed depending on the polymer and APIs used. The electrospun mats, based on PVP and KVA, were characterized by a soft and cotton–wool-like structure, whereas the mats composed of the E100 were more compact and brittle.

The PVP-containing fibers with LPV were characterized by a cylindrical shape and smooth surfaces, without beads on the string formation (Figure 1). The KVA-based fibers were less uniform and more tangled than the PVP-containing fibers. For the KVA- and Eudragit-based fibers, only the formulations with 20% of LPV contained no beads on the string formation. Compared to the fibers with RTV, the fibers containing LPV were characterized by a larger diameter, smoother, and less tangled structure (Figure 1 and Figure 2). The fibers containing 2.5% RTV based on PVP had smooth surfaces, although were more frayed than those containing 5% RTV (Figure 2). In the case of the KVA-based fibers, increasing the RTV content resulted in fibers with a more compact structure. For the KVA- and Eudragit-based fibers, bead formation was observed within the entire RTV concentration range. A greater number of highlights and beads in the string formation compared to the LPV fibers may be due to the different concentrations of APIs, resulting in lower viscosity of the electrospinning solutions. The Eudragit-containing fibers were tangled and characterized by the most brittle and compact structure of all polymers regardless of the APIs used (Figure 1 and Figure 2). Measurements of fiber diameter indicated that all the fibers obtained containing both LPV and RTV were characterized by heterogeneous thicknesses, resulting in large standard deviations from the mean diameter. It was observed that the diameter of the fibers changed with increasing concentrations of the polymer and APIs.

The PVP-based fibers containing LPV were characterized by diameters ranging from 978 ± 354 nm to 2583 ± 744 nm for the formulation with the lowest and highest LPV concentrations (for formulations containing 10 and 20% LPV, larger needles of 0.7 mm and 0.8 mm, respectively, were used). For the KVA-based fibers, the largest standard deviations from the fiber diameter mean values were observed. With the increasing API concentration, the fiber diameter ranged from 1208 ± 860 nm to 3446 ± 1855 nm. Similarly, the diameters of Eudragit-based fibers with LPV ranged from 1062 ± 674 nm to 1850 ± 633 nm. The thicknesses of the PVP-based fibers containing RTV were 845 ± 240 nm and 961 ± 324 nm for the formulations containing 2.5% and 5% RTV, respectively. For the fibers produced from KVA, the diameters were 908 ± 272 nm for 2.5% RTV and 1260 ± 567 nm for 5% RTV formulations (for the 5% RTV fibers, a needle with a diameter of 0.7 mm was used). The Eudragit-based fibers had the smallest diameters of all the formulations produced, amounting to 635 ± 170 nm and 738 ± 281 nm for 2.5% and 5% RTV concentrations, despite using larger needles of 0.8 mm and 0.9 mm, respectively.

The fibers with the largest diameters were obtained from the 35% solution of KVA, whereas Eudragit-based fibers (25% solution) had the smallest diameter. In addition, regardless of the polymer used, an increase in fiber diameter was noted depending on the concentration of the therapeutic substance [40]. However, for some formulations, this may have been due to the use of larger needle diameters, as reported by Abdelhakim et al. [41].

### 3.2. Differential Scanning Calorimetry (DSC)

**Figure 3:**
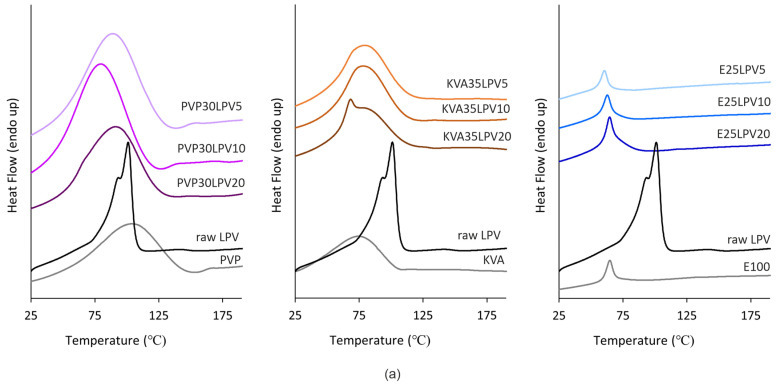

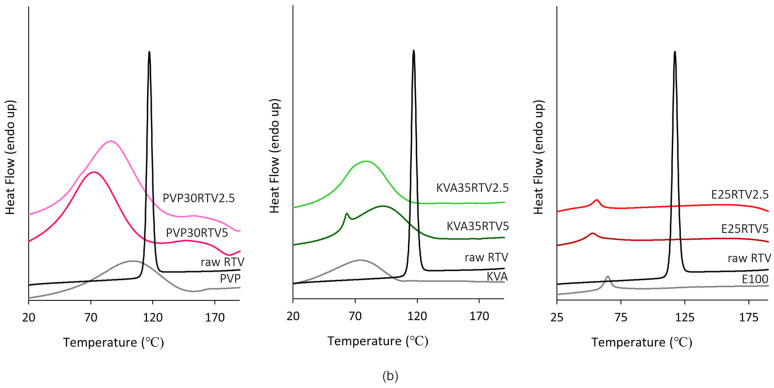
The DSC profiles of (**a**) electrospun fibers with LPV based on the PVP (purple lines), KVA (orange lines), and E100 (blue lines); (**b**) electrospun fibers with RTV based on PVP (pink lines), KVA (green lines), E100 (red lines), raw APIs (black line), and polymers (gray lines); measurement in the 1st run of heating.

The thermograms of raw LPV and RTV (black lines) are depicted in Figure 3a,b, respectively. As can be seen in Figure 3a, the raw LPV reveals a broad endothermic event associated with the melting of the LPV with an onset at 80 °C and two maxima at 93 °C and 100 °C [4,8,9]. For the raw RTV, clear, a single, sharp endothermic peak with an onset at 123 °C (Figure 3b) corresponds to the melting of the crystalline drug and is in agreement with the literature data [9,42].

The DSC curves of the neat polymers (Figure 3) are shown as gray lines. Regarding the PVP and KVA, the occurrence of a broad endothermic peak located in the range of 57–143 °C and 37–105 °C, respectively, can be linked to water evaporation. For PVP, a glass transition event was also observed with an onset at 156 °C [17]. In the case of E100, an endothermic peak at 58 °C can be identified on the DSC thermogram, corresponding to the glass transition [8,43].

In the analysis of the thermograms of the electrospun fibers based on the PVP and E100 as depicted in the Figure 3, the absence of a melting point indicated full amorphization of both LPV and RTV, regardless of their content. In the case of the PVP fibers with LPV (Figure 3a) and RTV (Figure 3b), two endothermic events are visible. The first with an onset at ca. 50 °C and 41 °C, respectively, can be linked to water evaporation, while the second one with an onset above 125 °C corresponds to the glass transition temperature of the polymer. The thermograms of the electrospun fibers based on the E100 indicate that an endothermic event with an onset temperature of 55 °C in the case of formulations with LPV and 42 °C and 50 °C for E25RTV5 and E25RTV2.5, respectively, is associated with the glass transition of the polymer. Regarding the KVA formulation, the broad endothermic events located in the range of 46–114 °C for the LPV formulations and 52–126 °C for the RTV fibers associated with water evaporation can be distinguished. However, as can be seen in the case of KVA35LPV20 and KVA35RTV5 (Figure 3), another peak is interposed on the broad thermal event with the midpoint temperature at 78 °C and 91 °C, respectively. This might be connected with the melting of the drugs, which indicates only their partial amorphization; however, further analysis is needed.

**Figure 4:**
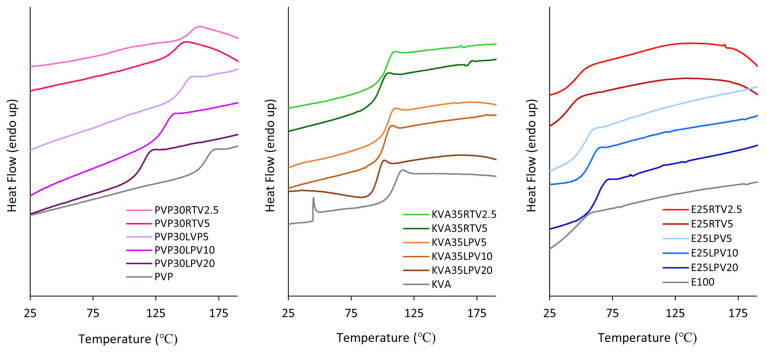
The DSC thermograms of the neat polymers and electrospun fibers with LPV and RTV measured in the 2nd run of heating.

Due to the presence of water in the polymers, particularly PVP and KVA [17,44], as well as in the prepared formulations, which may cover other thermal events, all samples were cooled down and then re-heated. Based on the thermograms obtained in the second run of heating, the temperature of the glass transition was determined as depicted in Figure 4. As can be seen, the onsets of the glass transition temperature of the fibers containing both LPV and RTV are located near the glass transition temperatures of the corresponding neat polymers. Therefore, it can be concluded that the glass transition event originates from the amorphous polymer. Similar findings were reported for solid dispersions with bicalutamide based on the poloxamers [22].

### 3.3. X-ray Diffraction (XRD)

**Figure 5:**
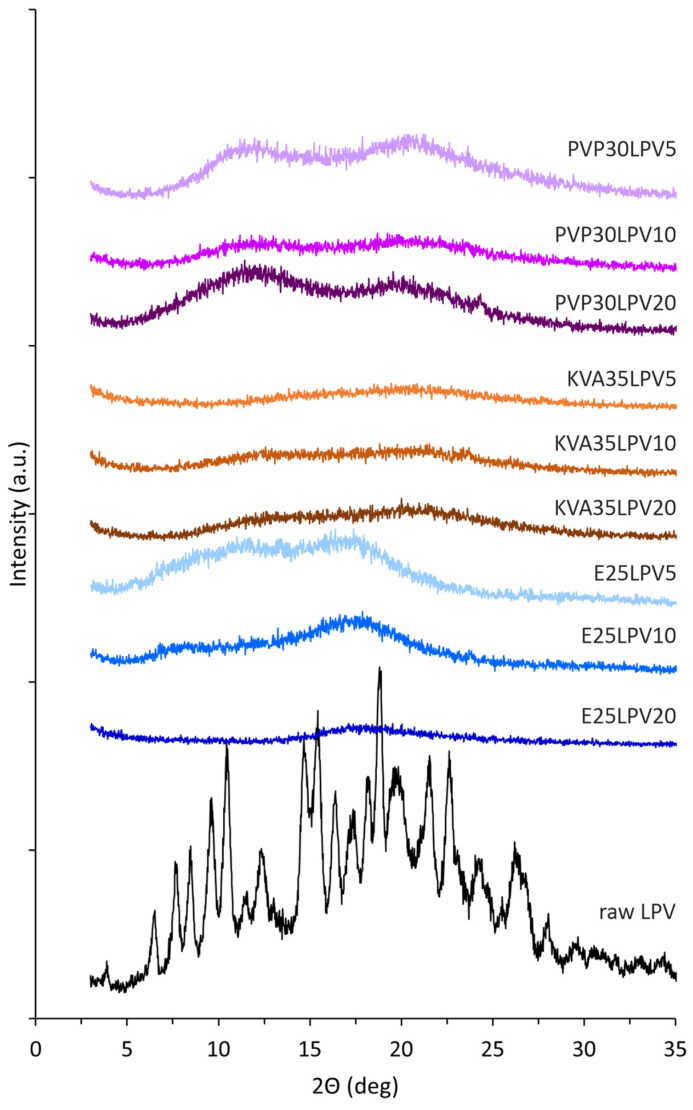
X-ray diffraction patterns of electrospun fibers containing LPV and raw LPV.

**Figure 6:**
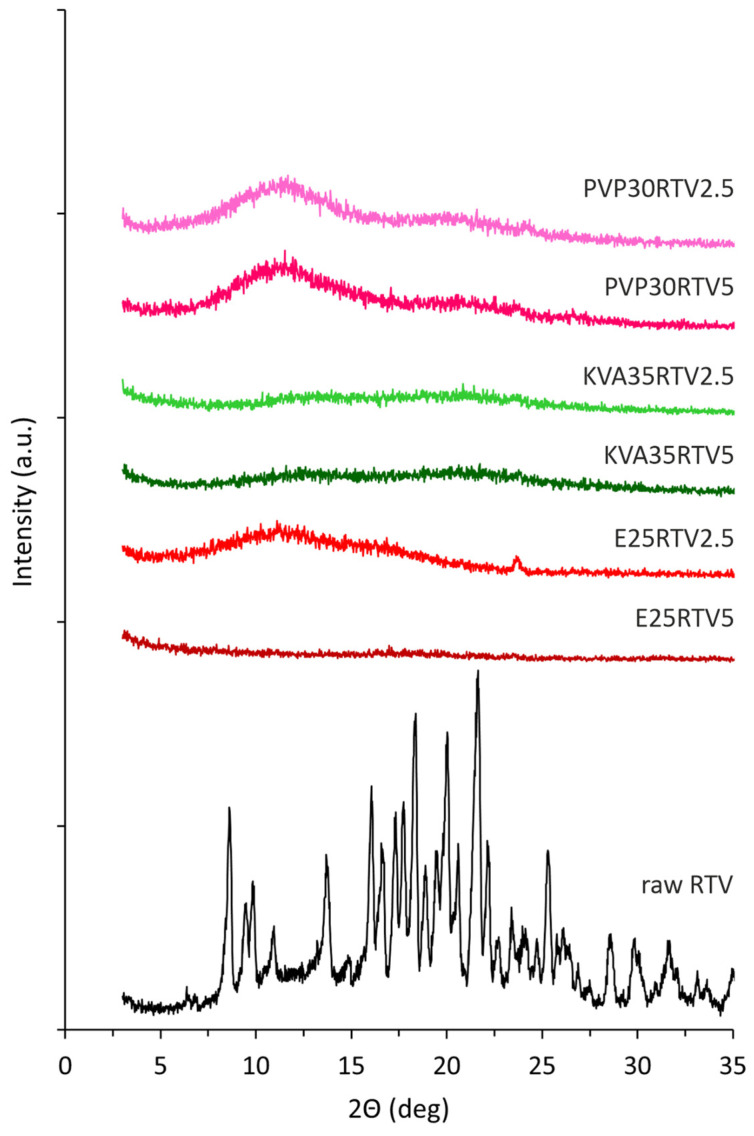
X-ray diffraction patterns of electrospun fibers containing RTV.

An XRD analysis was performed to assess the impact of the electrospinning process on the molecular structure of the model drug substances and confirm the amorphous nature of the prepared electrospun solid dispersions. The obtained diffractogram of the raw LPV (Figure 5) with the sharp Bragg peaks at 2θ values of 6.5°, 7.7°, 12.3°, 14.7°, 15.4°, 16.4°, 18.8°, 19.6°, 21.5°, 22.7°, and 26.3° confirms its crystalline structure. A comparable diffraction pattern for lopinavir was described in the literature [4,5,8,45]. Ritonavir has three polymorphic forms, which differ in solubility, dissolution rate, and bioavailability [42,46,47,48]. The presence of the sharp, crystalline Bragg peaks on the diffractogram pattern for raw RTV (Figure 6) at 6.4°, 8.6°, 9.5°, 9.8°, 10.9°, 13.7°, 16.1°, 18.3°, 20.0°, 21.6°, and 22.2° indicates the occurrence of polymorphic form II, which is confirmed by literature data [42].

As can be seen in Figure 5 and Figure 6, the presence of the characteristic amorphous halo in the diffractograms collected for electrospun fibers containing both LPV and RTV indicates full amorphization, regardless of the API content and type of polymeric matrix. The differences in the results obtained from DSC measurement for KVA35LPV20 and KVA35RTV5, where thermograms reveal the occurrence of a crystalline fraction in the samples, may be due to the discrepancy resulting from the different analytical procedures and sensitivity of both methods.

### 3.4. Wettability Study

The wetting properties of the raw APIs and investigated fibers were evaluated by water contact angle measurements. This parameter plays an important role in pharmaceutical sciences due to its impact on drug dissolution, solubilization, and disintegration of a dosage form [18,22,49,50,51].

**Figure 7:**
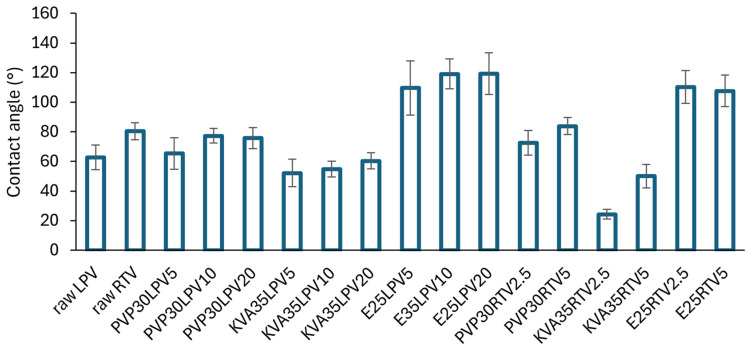
The values of contact angles of raw LPV, raw RTV, and electrospun fibers containing LPV and RTV.

The results of the contact angle analysis are presented in Figure 7. The type of polymer used as a carrier in solid dispersions had an impact on the contact angle values. Both PVP and KVA are hydrophilic polymers with contact angles of approx. 40° [49,50], and Eudragit 100 is characterized by hydrophobic properties (θ > 100°) [52]. The fiber wettability results reflected these features. Regardless of the type of the incorporated drug substance, the contact angle values of the PVP and KVA formulations were below 90°, whereas they exceeded 110° in the case of the E100-based fibers. Moreover, the water droplet immediately penetrated the hydrophilic fibers, while in the case of hydrophobic fibers, it remained on the surface throughout the time of the test. However, only the KVA systems with contact angle values ranging between 52–60° and 24–50° for the LPV and RTV formulations, respectively, showed improved wettability compared to the raw APIs (θ_LPV_ = 62.7 ± 8.3°, θ_RTV_ = 80.4 ± 5.8°). Furthermore, it was found that the higher the drug content in the formulations, the higher the value of the contact angle.

### 3.5. Drug Loading (DL) and Encapsulation Efficiency (EE%)

Electrospinning is indicated as a process enabling the preparation of nanofibers characterized by high loading capacity and high encapsulation efficiency [53,54]. The drug content varied between formulations due to the different dry mass of ingredients in electrospun solutions. The amount of the APIs dissolved was the same for all formulations, i.e., 5.0%, 10.0%, and 20% or 2.5% and 5.0% for the LPV and RTV formulations, respectively, so the concentration of the polymer solution was different (Table 1). Generally, the lower the polymer concentration, the higher the drug content. The API content in the investigated fibers ranged from approx. 12% to 49% and 7% to 18% of dry mass for the LPV and RTV formulations, respectively (Table 3).

The measured LPV and RTV loading was close to the theoretical content, which was confirmed by high EE% values between 86% and 101% (Table 3). Most of the investigated fibers were also characterized by a low RSD below 5%, which indicates homogeneous API dispersion in the polymeric matrix. Only in the case of the formulation E25LPV5 do the obtained results, i.e., EE% over 106% and RSD 10.6%, suggest heterogeneous dispersion of LPV in the fibers.

### 3.6. Equilibrium Solubility Study

**Figure 8:**
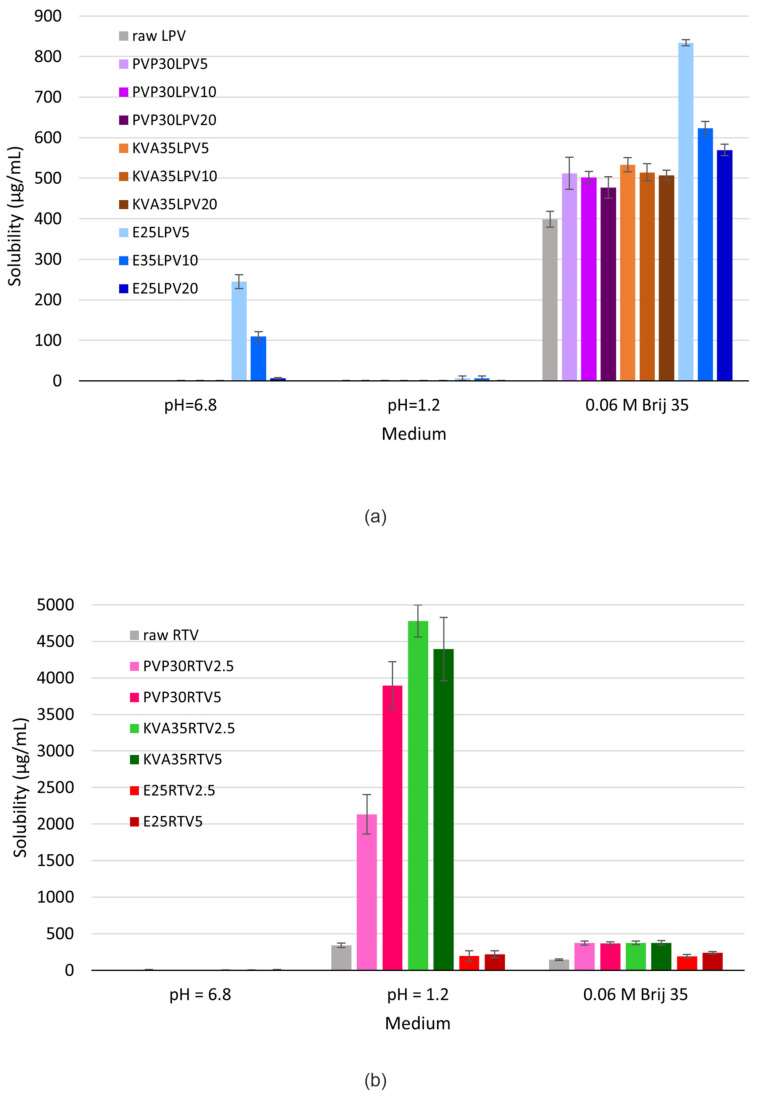
Solubility data of (**a**) raw LPV and fibers with LPV; (**b**) raw RTV and fibers with RTV.

The equilibrium solubility studies were performed for the raw APIs and all investigated fibers in phosphate buffer (pH = 6.8), 0.1 M HCl (pH = 1.2), and in the medium recommended by the USP 43-NF38 guidance for LPV/RTV tablets [28], i.e., 0.06 M Brij-35 (pH = 4.1). The results obtained for both the LPV and RTV fibers show the differences between the solubilizing efficacy of the utilized polymers. The results from the saturation solubility tests for the LPV and RTV formulations are presented in Figure 8.

The physicochemical properties of LPV (Table 2) and published data [55] indicate that its solubility is independent of the pH of the medium. In the case of the LPV formulations, the highest solubility improvement in the phosphate buffer and Brij solution was achieved using E100 as a polymeric matrix, while in the acidic conditions the solubility values for all formulations were at a low level. Furthermore, as shown in Figure 8a, the LPV solubility from investigated fibers increased with decreasing API content. Among all investigated formulations, E25LPV5 exhibited the highest solubility in the tested media (phosphate buffer and Brij-35 solution), i.e., 244.8 ± 16.7 µg/mL and 833.9 ± 7.9 µg/mL, respectively. This was 1224- and 2-fold higher in comparison with the crystalline LPV. The obtained results, in particular the significant improvement in solubility in an alkaline environment, were surprising, since based on the data from the literature [43,56,57], Eudragit^®^ E is primarily useful for improving solubility in acidic pH due to its good solubility at a pH below 5.0. At a higher pH, it is swellable and permeable [58], which could explain the increased solubility in an alkaline environment.

In contrast, the highest solubility for the RTV formulations was obtained in acidic conditions, due to its alkaline nature (Table 2), which is in accordance with the findings reported by Trasi et al. [55]. The best solubility improvement was obtained using KVA as a carrier. The solubility of raw RTV was found to be 340.4 ± 31.9 µg/mL, while the KVA-based formulations were more than 13-fold higher (Figure 8b). In the Brij-35 solution at pH = 4.1, the PVP- and KVA-based solid dispersions were similar in solubility, i.e., approx. 370 µg/mL, which was only 2.6-fold greater than the crystalline RTV. However, this medium was selected for further dissolution studies because it provided favorable conditions for both LPV and RTV.

### 3.7. Dissolution Study

**Figure 9:**
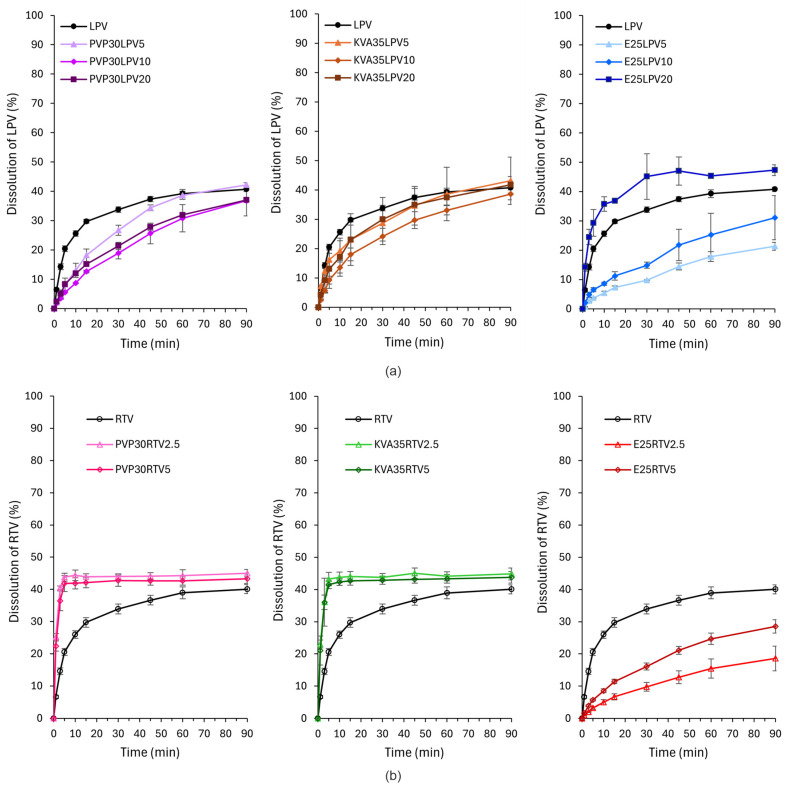
Dissolution profile of the electrospun fibers containing (**a**) LPV and (**b**) RTV.

Dissolution studies were performed to examine the impact of the type of polymeric carrier and drug loading on the dissolution rate. The dissolution profiles are presented in Figure 9. For the LPV formulations, only E25LPV20 exhibited a faster dissolution rate compared to the raw APIs. The amount of LPV dissolved after 1 and 30 min was approx. 2- and 1.3-fold higher than in the case of bulk drug substances, respectively (Figure 9a). In the case of the remaining formulations, the amount of the dissolved drug was lower or comparable to raw LPV. Surprisingly, the slowest API dissolution was reported for E25LPV5, where the amount of LPV released after 90 min was 1.5-fold lower than for the crystalline LPV. The dissolution test results are in contradiction with the results from the equilibrium solubility studies (Figure 8a). This inconsistency may be due to the timing of both tests; however, further analysis will be performed to explain this phenomenon.

In contrast to the results for LPV, the greatest improvement in the dissolution rate for fibers with RTV was shown for fibers based on PVP and KVA, regardless of the API content (Figure 9b). In both cases, the amount of RTV dissolved after 1 and 5 min was more than 3- and 2-fold higher in comparison to the crystalline RTV. The E100 fibers exhibited slower dissolution rates than the bulk RTV, and similarly to the LPV systems, the lower the drug content, the slower the dissolution. After 90 min, the amount of the drug released from E25RTV5 and E25RTV2.5 was 1.4- and 2.2- fold lower compared to the raw APIs. The increased dissolution rate of RTV from PVP- and KVA-based fibers may be related to their improved wettability and thus facilitated water penetration through the polymer matrix [18,59].

## 4. Conclusions

The results prove that the use of the electrospinning process enables the preparation of ASDs containing LPV and RTV, characterized by high encapsulation efficiency, increased solubility, and an increased dissolution rate. The selection of the appropriate polymeric carrier as well as API concentration in electrospun solutions is a key factor, which impacts the ASD properties. In this work, all investigated polymers led to the amorphization of both the LPV and RTV, which was confirmed in DSC and XRD studies. However, based on the solubility and dissolution test results, for the LPV and RTV formulations, the most promising polymers were Eudragit^®^ E100 and Kollidon^®^ VA64, respectively, despite the bead-on-fiber morphology and their heterogeneous thickness. In the case of the E100-based fibers with LPV, formulations with 5% and 20% of the drug substance featured the highest solubility and dissolution rate, respectively. In turn, for the RTV formulations, the use of KVA as a polymeric carrier resulted in the greatest improvement in both solubility and the dissolution rate. In summary, E25LPV20 and KVA35RTV5 were selected as the most promising formulations.

Both investigated APIs are used for antiretroviral therapy in children and are listed on the World Health Organization 2023 Model List of Essential Medicines for Children [60]. Therefore, taking into account the obtained results, further studies will include the development of pediatric forms of the drug, such as orodispersible films and minitablets and their long-term stability.

## Figures and Tables

**Table 1:** Composition of electrospinning solutions.

Formulation	Content of Electrospinning Solution Components (%)				
	30% PVP	35% KVA	25% E	LPV	RTV
PVP30LPV5	95.0	-	-	5.0	-
PVP30LPV10	90.0	-	-	10.0	-
PVP30LPV20	80.0	-	-	20.0	-
KVA35LPV5	-	95.0	-	5.0	-
KVA35LPV10	-	90.0	-	10.0	-
KVA35LPV20	-	80.0	-	20.0	-
E25LPV5	-	-	95.0	5.0	-
E25LPV10	-	-	90.0	10.0	-
E25LPV20	-	-	80.0	20.0	-
PVP30RTV2.5	97.5	-	-	-	2.5
PVP30RTV5	95.0	-	-	-	5.0
KVA35RTV2.5	-	97.5	-	-	2.5
KVA35RTV5	-	95.0	-	-	5.0
E25RTV2.5	-	-	97.5	-	2.5
E25RTV5	-	-	95.0	-	5.0

**Table 2:** Physicochemical properties of the model drugs [36,37,38,39].

Properties	Lopinavir	Ritonavir
Molecular weight	628.8 g/mol	720.9 g/mol
Water solubility	1.92 µg/mL	1.26 µg/mL
Ethanol solubility	freely soluble	freely soluble
logP	4.69	5.22
pKa (strongest acidic condition)	13.39	13.68
pKa (strongest basic condition)	−1.5	2.84

**Table 3:** DL and EE% of the electrospun fibers containing LPV or RTV (*n* = 3).

Formulation	DL ± SD (% *w/w*)	EE% ± RSD (%)
PVP30LPV5	12.9 ± 0.3	86.9 ± 2.6
PVP30LPV10	23.7 ± 0.7	87.6 ± 3.2
PVP30LPV20	39.7 ± 1.3	87.2 ± 3.8
KVA35LPV5	12.2 ± 1.0	93.5 ± 8.8
KVA35LPV10	22.6 ± 0.1	94.0 ± 0.3
KVA35LPV20	37.8 ± 0.3	90.7 ± 1.0
E25LPV5	18.5 ± 2.1	106.1 ± 10.6
E25LPV10	23.0 ± 0.5	97.3 ± 1.7
E25LPV20	49.2 ± 1.3	98.5 ± 2.7
PVP30RTV2.5	7.5 ± 0.2	94.7 ± 2.7
PVP30RTV5	14.0 ± 0.3	93.6 ± 2.0
KVA35RTV2.5	6.8 ± 0.1	100.4 ± 1.9
KVA35RTV5	12.6 ± 0.2	96.1 ± 1.8
E25RTV2.5	9.4 ± 0.1	101.0 ± 0.4
E25RTV5	17.6 ± 0.4	101.4 ± 2.4

## Data Availability

The original contributions presented in the study are included in the article, further inquiries can be directed to the corresponding authors.
